# Differential modulation of sensorimotor alpha rhythms by motor imagery, electrical stimulation, and mirror visual feedback following peripheral nerve reconstruction: an EEG study

**DOI:** 10.3389/fneur.2026.1836039

**Published:** 2026-08-21

**Authors:** Yueh-Hsia Chen, Shih-Heng Chen, Cheng-Hung Lin

**Affiliations:** 1 School and Graduate Institute of Physical Therapy, College of Medicine, National Taiwan University, Taipei, Taiwan; 2 Division of Physical Therapy, Department of Physical Medicine and Rehabilitation, National Taiwan University Hospital, Taipei, Taiwan; 3 Department of Plastic and Reconstructive Surgery, Linkou Chang Gung Memorial Hospital, Taoyuan, Taiwan; 4 College of Medicine, Chang Gung University, Taoyuan, Taiwan; 5 Center for Vascularized Composite Allotransplantation, Department of Plastic and Reconstructive Surgery, Chang Gung Memorial Hospital, Taoyuan, Taiwan

**Keywords:** cortical plasticity, electrical stimulation (ES), electroencephalography, mirror visual feedback (MVF), motor imagery (MI), neurorehabilitation, peripheral nerve injury, sensorimotor rhythm

## Abstract

**Objectives:**

Peripheral nerve reconstruction often requires prolonged rehabilitation to restore sensorimotor function. Motor imagery (MI), electrical stimulation (ES), and mirror visual feedback (MVF) are commonly used rehabilitation strategies; however, their comparative effects on cortical oscillatory dynamics during early neural recovery remain unclear. This study investigated EEG sensorimotor rhythm modulation during MI, ES, and ES combined with MVF in individuals who had undergone upper limb peripheral nerve reconstruction.

**Methods:**

Eleven patients, within six months after peripheral nerve reconstruction, and twenty-one age-matched healthy participants were recruited. Electroencephalography was recorded during the resting state and three independent task conditions: MI, ES with sham MVF, and ES combined with MVF. Relative power (RP) and baseline-normalized changes in relative power (ΔRP) in EEG spectral bands were calculated to characterize task-related modulation of sensorimotor rhythms. To control for multiple condition contrasts, *p* values were adjusted using the Benjamini–Hochberg false discovery rate (FDR-adjusted) procedure within each frequency band and group.

**Results:**

Task-related modulation was most pronounced in the alpha band. In healthy participants, alpha RP significantly decreased during all task conditions compared with the resting state (all FDR-adjusted *p* < 0.001). In contrast, patients showed significant alpha RP suppression during ES and MVF (FDR-adjusted *p* < 0.001 and *p* = 0.002, respectively), whereas MI alone showed less evident alpha RP suppression and did not reach statistical significance after FDR correction (FDR-adjusted *p* = 0.078). Greater alpha band ΔRP during MVF than MI was observed in healthy participants (FDR-adjusted *p* = 0.009), whereas the corresponding contrast did not remain significant in patients after FDR correction (FDR-adjusted *p* = 0.090). Modulation of beta rhythms was limited in both groups (all FDR-adjusted *p* > 0.05).

**Conclusion:**

These findings suggest that alpha modulation during MI was less evident in patients following peripheral nerve reconstruction. However, given the limited sample size, the non-significant MI finding should be interpreted cautiously. In contrast, ES-based interventions, particularly when combined with MVF, were associated with greater sensorimotor rhythm modulation, supporting the potential role of multimodal rehabilitation strategies in facilitating cortical reorganization during early neural recovery after peripheral nerve repair.

## 1 Introduction

Peripheral nerve injury induces substantial structural and functional reorganization within the central nervous system, including both anterograde and retrograde degeneration (1, 2). Although reversed cortical reorganization has been reported following nerve transfer, hand replantation, and transplantation (3, 4), incomplete sensorimotor recovery and prolonged nonuse remain common after deafferentation, posing significant challenges to functional restoration (5). Therefore, early interventions targeting cortical plasticity are essential to prevent maladaptive reorganization and to preserve sensorimotor representations of the affected limb (6).

Motor simulation interventions, including motor imagery (MI), action observation, and mirror therapy, have been widely demonstrated to facilitate motor recovery following peripheral nerve repair (7), reduce pain (8), and enhance motor learning in both healthy individuals and patients (9). These effects are thought to be mediated by the recruitment of mirror neuron systems (10) and increased sensorimotor cortical engagement. Neurophysiologically, such engagement is often reflected by task-related suppression of alpha (8–12 Hz) and beta (12–30 Hz) oscillations in electroencephalography (EEG), which are established markers of sensorimotor activation (11, 12). While reduced alpha and beta power during motor simulation has been reported in stroke populations (13), its manifestation following peripheral nerve reconstruction remains unclear.

In parallel, neuromuscular electrical stimulation (ES) has been shown to induce sensorimotor cortical plasticity (14) and promote peripheral nerve regeneration through molecular mechanisms resembling natural recovery processes (15, 16). Despite its widespread clinical use, the effects of ES on cortical oscillatory dynamics in individuals with peripheral nerve injury are still insufficiently characterized.

Although MI, ES, and mirror visual feedback (MVF) have each been shown to modulate sensorimotor cortical activity, their comparative effects on cortical oscillatory dynamics following peripheral nerve reconstruction remain largely unknown. In particular, little is known about how these rehabilitation strategies influence sensorimotor rhythm modulation during the early stage of neural recovery.

Therefore, the present study aimed to investigate task-related modulation of EEG spectral power during MI, ES, and MVF in individuals who had undergone upper limb peripheral nerve reconstruction. Relative power (RP) and baseline-normalized changes in relative power (ΔRP) were analyzed to characterize sensorimotor rhythm modulation and to compare these responses with those observed in healthy individuals.

Based on previous evidence demonstrating task-related suppression of sensorimotor rhythms during MI and action observation paradigms, we further hypothesized that sensorimotor modulation in this population would predominantly manifest within the alpha and beta frequency bands. In addition, we expected that MVF would elicit stronger suppression of sensorimotor rhythms compared to MI alone, reflecting enhanced engagement of the sensorimotor network during multimodal rehabilitation.

## 2 Materials and methods

### 2.1 Study design and participants

This research is a case–control study. The participants were enrolled from a university research laboratory. Sample size estimation was performed using G*Power (version 3.1.9). The *a priori* effect size assumption (Cohen’s d = 0.8) was based on previous neurophysiological studies reporting large task-related modulation of sensorimotor cortical activity during MI and MVF paradigms (17, 18). This estimate referred to expected task-related modulation of sensorimotor rhythm power (RP) across the experimental conditions, rather than a specific between-group comparison. With an alpha level of 0.05 and statistical power of 0.80, the minimum required sample size was estimated to be 10 patients. Accordingly, 11 participants who had undergone upper limb peripheral nerve reconstruction within the previous 6 months were recruited. Additionally, 21 age-matched healthy participants (7 males, mean age 33.2 ± 12.4 years) were enrolled from a national university and hospital as controls. Healthy participants were included to provide a reference for normal sensorimotor rhythm modulation.

Participants were excluded if they had any central nervous system disorders, had history of latent neuropathy (e.g., diabetes, dialysis), or had difficulty following instructions. Additionally, all participants were required to score an average of 2 or higher on the Kinesthetic and Visual Imagery Questionnaire-10 for kinesthetic imagery (19); those who did not meet this criterion were excluded. As 2 participants felt that the EEG hat induced headache, finally 9 patients (8 males, mean age 35.6 ± 11.5 years) completed the experiment. The demographic and characteristic data are shown in Table 1.

**Table 1:** Demographic and clinical characteristics of the study participants.

Characteristic	Patients (*n* = 9)	Healthy subjects (*n* = 21)
Age (years), mean (SD)	35.6 (11.5)	33.2 (12.4)
Sex, *n* males (%)	8 (88.9)	7 (33.3)
Right-hand dominant (%)	9 (100)	20 (95.2)
Right-hand injury (%)	4 (44.4)	
Days after surgery (day), mean (SD)	76 (44.8)	
Injured nerve (%)		
Brachial plexus	1 (11.1)	
Median and ulnar	1 (11.1)	
Median	1 (11.1)	
Ulnar	6 (66.7)	
Location of injury (%)		
Neck	1 (11.1)	
Upper arm	1 (11.1)	
Forearm	7 (77.8)	
Type of nerve injury (%)		
Cutting	6 (66.7)	
Avulsion	1 (11.1)	
Traction	1 (11.1)	
Compression	1 (11.1)	
Operation (%)		
Repair	5 (55.6)	
Nerve graft	2 (22.2)	
Nerve transfer	2 (22.2)	
Semmes Weinstein monofilament test (evaluator size at thenar or hypothenar) (%)		
Undetectable	2 (22.2)	
6.65	1 (11.1)	
4.56	5 (55.6)	
4.31	1 (11.1)	

Participants were instructed to abstain from caffeine and alcohol, clean their hair the previous night, and have enough sleep time before the experiment. This study was conducted in accordance with the Declaration of Helsinki, approved by Research Ethics Committee of National Taiwan University Hospital (NO: 202309076RINA), and registered on ClinicalTrial.gov (NCT06209801). Before participation, informed consent was obtained from all participants. This study was reported in accordance with the STROBE guidelines.

### 2.2 Experimental procedures

**Figure 1:**
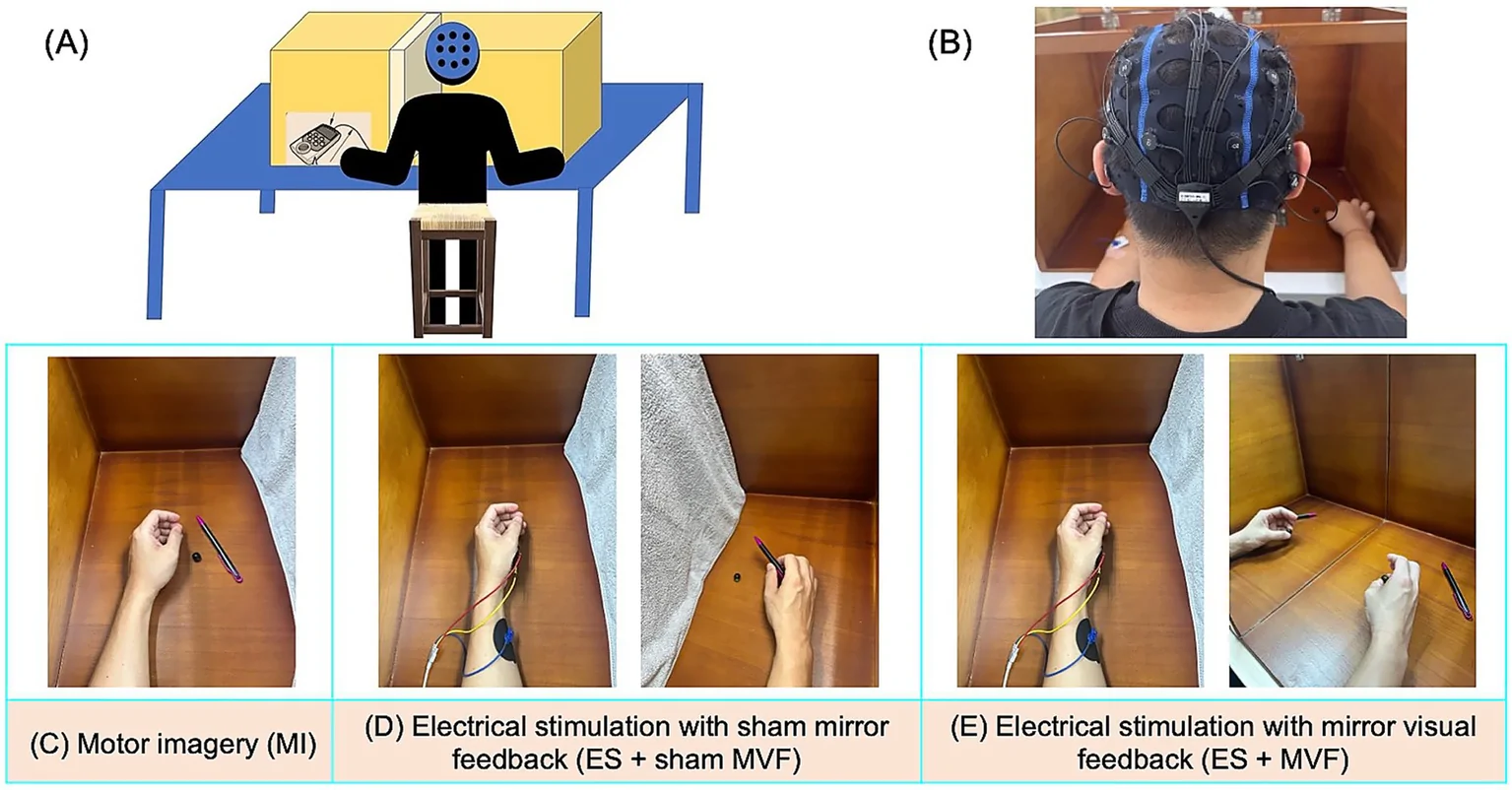
Experimental paradigm. **(A)** Schematic of the experimental design. Participants were seated in front of a mirror box while EEG signals were recorded from sensorimotor cortical regions. **(B)** EEG was recorded from the C3 and C4 electrodes according to the International 10–20 system to assess sensorimotor cortical activity. **(C)** In the MI (motor imagery) condition, participants kinesthetically imagined using their non-dominant (healthy subjects) or injured (patients) fingers to pick up a pen and a marble. **(D)** In the ES condition, electrical stimulation was applied to the non-dominant or injured hand while participants viewed their dominant or non-affected hand performing the task through a sham mirror box. No mirror visual feedback or explicit motor imagery instructions were provided during this condition. **(E)** In the MVF (mirror visual feedback) condition, electrical stimulation was similarly applied, but participants observed the mirror reflection of their dominant or non-affected hand as if it were their non-dominant or injured hand performing the task.

Three experimental conditions included: (1) MI alone (MI); (2) ES without MVF (ES); and (3) ES combined with MVF (MVF) were randomly assigned to each participant. Each participant sat in front of a mirror box with one hand on each side (Figure 1). The participant was informed of the experiment procedures and requested to minimize additional physical movement of other body parts throughout the EEG recording process.

All participants underwent a single EEG recording session. To ensure procedural consistency across participants, six versions of PowerPoint presentations were developed to guide the experimental conditions, with randomized sequences tailored for both patient and control groups. Prior to the recording session, all participants practiced the assigned tasks until they were familiar with the required movement patterns and pacing. Prior to recording, participants practiced the tasks until they were familiar with the required movement patterns and pacing. A one-minute resting-state EEG baseline was recorded at the beginning of the experiment. Each experimental condition lasted 60 s and was separated by a 30-s rest interval.

In the MI condition, participants sat facing their non-dominant (for healthy controls) or injured (for patients) hand. They were instructed to kinesthetically imagine performing two actions sequentially: (1) using the thumb and index finger to pick up a pen 10 times and (2) using the thumb and ring finger to pick up a marble 10 times, all within a 60-s timeframe. Before EEG recording, participants completed a brief practice session to ensure understanding of the kinesthetic MI task. During task performance, participants were visually monitored by the investigator to minimize overt movements and compensatory muscle activity.

In the ES condition, participants observed their dominant or unaffected hand performing the same actions, without mirror visual feedback. No explicit MI instructions were provided during this condition. In the MVF condition, participants observed the mirror reflection of their dominant or unaffected hand, creating the visual illusion that the non-dominant or affected hand was performing the movements. In both the ES and MVF conditions, neuromuscular electrical stimulation (30 Hz with a current of 10 mA) was applied to the target muscle of the non-dominant hand (healthy participants) or the affected hand (patients).

### 2.3 EEG data acquisition and processing

EEG was recorded using a 24-channel wireless EEG system (Smart BCI 24, Mitsar Co. Ltd., Russia) with a sampling rate of 250 Hz, maintaining electrode impedance below 5 kΩ. Electrodes were positioned according to the International 10–20 system at C3 and C4, while Fp1 and Fp2 detected eye movements and blinking activity. A band-pass filter (0.5–30 Hz) and a notch filter (60 Hz) were applied. Analyses were restricted *a priori* to C3 and C4 because these electrodes overlie the bilateral sensorimotor cortices and are commonly used to evaluate sensorimotor rhythm modulation during MI and MVF paradigms. EEG signals were acquired and processed using EEGStudio software (Mitsar Co. Ltd., Russia). Biological artifacts, such as eye blinks and muscle activity, were removed using independent component analysis (ICA) (20).

Since absolute EEG spectral power varies across individuals, RP was used to normalize band power (21). RP was selected because it normalizes inter-individual variability in EEG amplitude and has been widely used to characterize sensorimotor rhythm modulation during motor imagery and neurorehabilitation paradigms. The RP of each frequency band was calculated to normalize individual variability in EEG spectral power, as shown in the following formula.

$$\mathrm{RP}=\frac{\text{absolute power of each spectral band}}{\text{absolute power of total spectral bands}}\times 100\%$$

Each experimental condition consisted of a continuous 60-s EEG recording, which was analyzed as a single condition-level segment after preprocessing. After filtering and ICA-based artifact correction, condition-level spectral power values were extracted for each frequency band. RP was calculated from these condition-level spectral estimates and used for subsequent statistical analyses. Because the study focused on sustained task-related spectral modulation rather than event-locked responses, no additional event-locked epoching was applied in the GEE analyses.

Given their distinct roles in the motor cortex, the beta band can be further divided into low beta (<20 Hz) and high beta (>20 Hz) frequencies (22, 23), with the low beta band associated with reaction time and the high beta band with attention. Alpha (8–12 Hz), low beta (12–20 Hz), and high beta (20–30 Hz) band powers were extracted from the non-dominant sensorimotor cortex in healthy subjects or the contralateral sensorimotor cortex in patients with peripheral nerve reconstruction. Then the ratio of a frequency band’s power to total band power was calculated for alpha, low beta, and high beta bands. To compare RP changes across conditions, the deviation of RP from baseline (∆RP) computed using the following formula (24, 25).

$$\mathrm{\Delta RP}=\frac{\mathrm{RP}-RP_{\text{baseline}}}{RP_{\text{baseline}}}$$

### 2.4 Statistical analysis

Statistical analyses were performed using Python (version 3.12.13) with the statsmodels package. Generalized estimating equation (GEE) models were employed to account for within-subject correlations in repeated measures data. GEE was selected due to its robustness in handling correlated observations, its ability to provide consistent parameter estimates under misspecification of the correlation structure, and its suitability for small sample sizes and unbalanced data (26). An exchangeable working correlation structure was specified, and robust sandwich standard errors were applied. Separate GEE models were constructed for each outcome variable, including RP and ΔRP. Analyses were conducted separately for each frequency band and within each group.

For RP analyses, the resting state was specified as the reference condition. For ΔRP analyses, ΔRP values were first calculated relative to the resting-state RP (baseline), and MVF was subsequently specified as the reference category in the GEE model solely for statistical comparisons among the intervention conditions. To control for multiple condition contrasts, *p* values were adjusted using the Benjamini–Hochberg false discovery rate procedure within each predefined GEE model. Specifically, correction was applied across the three condition contrasts in each RP model and across the two condition contrasts in each ΔRP model. Statistical significance was defined as an FDR-adjusted *p* < 0.05.

## 3 Results

### 3.1 Relative power of EEG spectral bands

**Figure 2:**
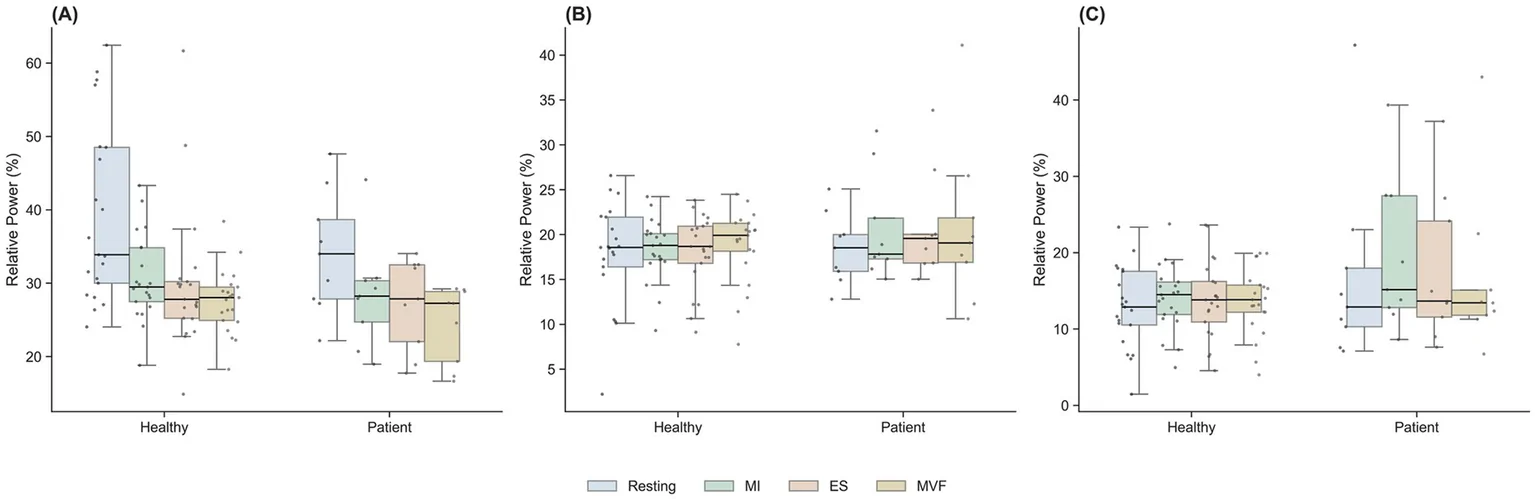
Relative power (RP) of sensorimotor rhythms during resting and task conditions. Boxplots show the distribution of RP values in healthy participants (*n* = 21) and patients following peripheral nerve reconstruction (*n* = 9). Boxes represent the interquartile range (IQR), center lines indicate the median, whiskers extend to 1.5 × IQR, and gray dots represent individual observations. **(A)** Alpha band. **(B)** Low beta band. **(C)** High beta band. Separate generalized estimating equation (GEE) models were fitted for healthy participants and patients to evaluate condition effects within each group, using the resting condition as the reference level. No between-group comparisons were performed. Benjamini–Hochberg false discovery rate (FDR) correction was applied within each predefined GEE model. Detailed statistical results are presented in Table 2. MI, motor imagery; ES, electrical stimulation with sham mirror visual feedback; MVF, electrical stimulation combined with mirror visual feedback.

RP of EEG spectral bands during the resting state and task conditions is presented in Figure 2 and Table 2. Task-related modulation was most evident in the alpha band. In healthy participants, alpha RP in the sensorimotor cortex significantly decreased during all task conditions compared with the resting state, including MI, ES, and MVF (all FDR-adjusted *p* < 0.001).

**Table 2:** Relative power of sensorimotor rhythms across experimental conditions.

Frequency band	Condition	Healthy subjects (*n* = 21)			Patients (*n* = 9)		
		Mean ± SE	B (95% CI)	FDR-adjusted *p* value	Mean ± SE	B (95% CI)	FDR-adjusted *p* value
Alpha	Resting	39.23 ± 2.64	Reference	–	34.14 ± 2.74	Reference	–
	MI	30.68 ± 1.27	−8.55 (−12.23, −4.87)	**<0.001**	28.33 ± 2.41	−5.81 (−12.28, 0.66)	0.078
	ES	30.00 ± 2.11	−9.23 (−13.82, −4.63)	**<0.001**	27.19 ± 2.08	−6.95 (−10.11, −3.79)	**<0.001**
	MVF	27.58 ± 0.95	−11.65 (−16.69, −6.62)	**<0.001**	24.41 ± 1.74	−9.73 (−15.76, −3.71)	**0.002**
Low beta	Resting	17.88 ± 1.26	Reference	–	18.41 ± 1.23	Reference	–
	MI	18.36 ± 0.75	0.48 (−1.71, 2.67)	0.875	19.23 ± 1.08	0.82 (−0.95, 2.59)	0.080
	ES	18.09 ± 0.90	0.21 (−2.46, 2.89)	0.875	19.50 ± 1.10	1.09 (0.37, 1.81)	0.080
	MVF	18.78 ± 0.90	0.90 (−1.64, 3.45)	0.875	18.12 ± 1.35	−0.29 (−2.22, 1.64)	0.300
High beta	Resting	12.87 ± 1.16	Reference	–	14.23 ± 1.94	Reference	–
	MI	14.05 ± 0.94	1.18 (−0.61, 2.96)	0.499	17.05 ± 1.92	2.82 (−0.83, 6.48)	0.654
	ES	13.87 ± 1.11	0.99 (−1.23, 3.22)	0.499	15.10 ± 1.78	0.87 (−1.64, 3.38)	0.783
	MVF	13.61 ± 0.96	0.74 (−1.41, 2.89)	0.499	13.72 ± 1.34	−0.50 (−2.25, 1.24)	0.783

Data are presented as mean ± standard error (SE). Generalized estimating equation (GEE) models were used to estimate condition effects relative to the resting state (reference). Regression coefficients (B), 95% confidence intervals (CI), and Benjamini–Hochberg false discovery rate (FDR)-adjusted *p* values are reported for each condition. FDR correction was performed within each predefined GEE model. Significant adjusted *p* values (*p* < 0.05) are shown in bold. MI, motor imagery; ES, electrical stimulation with sham mirror visual feedback; MVF, electrical stimulation combined with mirror visual feedback.

In contrast, patients following peripheral nerve reconstruction demonstrated significantly lower alpha RP during ES and MVF than during the resting state (FDR-adjusted *p* < 0.001 and *p* = 0.002, respectively), whereas MI alone did not significantly differ from the resting state (FDR-adjusted *p* = 0.078). These findings suggest that task-related modulation of alpha rhythms was preserved during conditions involving sensory feedback. Although alpha RP during MI was also lower than at rest in patients (B = −5.81, 95% CI -12.28 to 0.66), this effect did not remain statistically significant after FDR correction.

Modulation in beta frequency bands was relatively limited. Low beta RP showed no significant differences across conditions in either group after FDR correction (all FDR-adjusted *p* > 0.05). Although a trend toward increased low beta power during ES was observed in patients, this effect did not remain significant following correction for multiple comparisons (FDR-adjusted *p* = 0.080). Similarly, high beta RP showed no significant modulation across task conditions in either group (all FDR-adjusted *p* > 0.05) (Table 2).

### 3.2 Baseline-normalized changes in relative power

**Figure 3:**
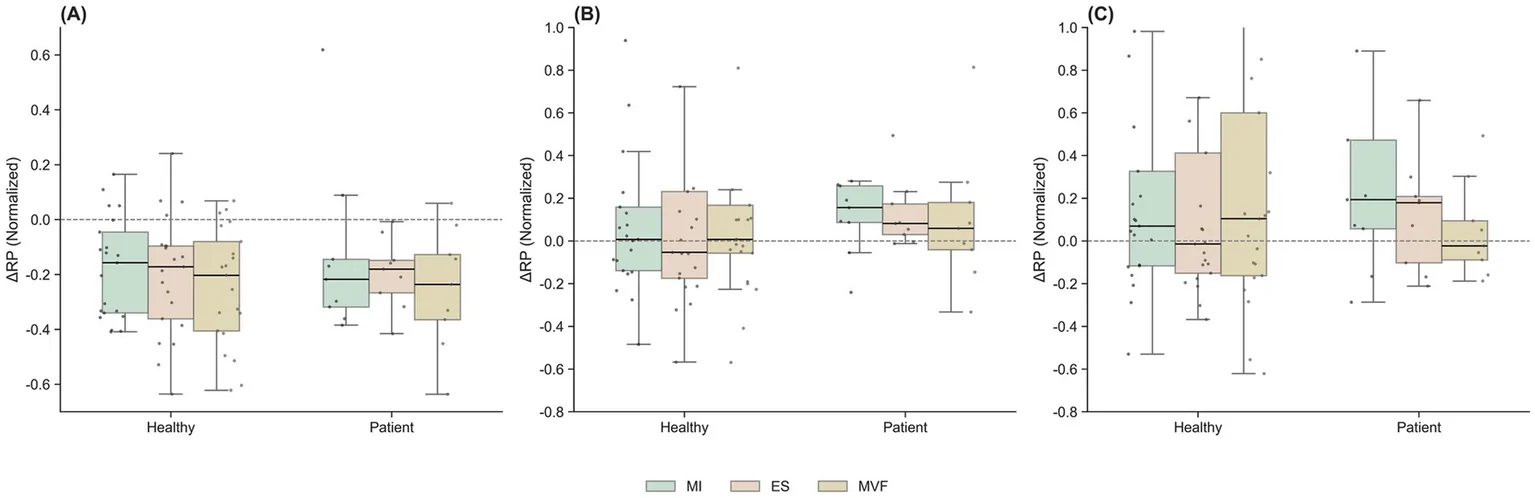
Baseline-normalized changes in relative power (ΔRP) across task conditions. ΔRP values represent task-related modulation of spectral power relative to the resting baseline. Boxplots show the distribution of ΔRP values in healthy participants (*n* = 21) and patients following peripheral nerve reconstruction (*n* = 9). Boxes represent the interquartile range (IQR), center lines indicate the median, whiskers extend to 1.5 × IQR, and gray dots represent individual observations. **(A)** Alpha band. **(B)** Low beta band. **(C)** High beta band. Separate generalized estimating equation (GEE) models were fitted for healthy participants and patients to evaluate condition effects within each group, using the mirror visual feedback (MVF) condition as the reference level. Benjamini–Hochberg false discovery rate (FDR) correction was applied within each predefined GEE model. Detailed statistical results are presented in Supplementary Table 1. MI, motor imagery; ES, electrical stimulation with sham mirror visual feedback; MVF, electrical stimulation combined with mirror visual feedback.

ΔRP are illustrated in Figure 3, and detailed model estimates are provided in Supplementary Table 1. In the alpha band, a significant difference between MI and MVF was observed only in healthy participants, with MVF producing a greater change in alpha RP than MI alone (FDR-adjusted *p* = 0.009). In patients, a similar trend was observed; however, the difference did not remain significant after FDR correction (FDR-adjusted *p* = 0.090). No significant differences were observed between ES and MVF in either group.

No significant differences in ΔRP were observed across task conditions in the low beta band in either group (all FDR-adjusted *p* > 0.05). Likewise, no significant differences were observed in the high beta band after FDR correction (all FDR-adjusted *p* > 0.05). Representative EEG spectral power patterns from one healthy participant and one patient are shown in Supplementary Figure 1.

## 4 Discussion

This study investigated the effects of neuromuscular ES, MI, and MVF on sensorimotor cortical activity following upper limb peripheral nerve reconstruction. Although even-related desynchronization (ERD) is commonly used to characterize task-related cortical activation (17), the present study employed RP and baseline-normalized changes in RP (ΔRP) to evaluate sensorimotor rhythm modulation during sustained task conditions. The principal findings were that alpha-band modulation was most pronounced during ES-based interventions. Both ES with sham MVF and ES combined with MVF induced significant alpha RP suppression in patients and healthy participants, whereas alpha modulation during MI alone was less evident in patients following peripheral nerve reconstruction. Furthermore, beta-band modulation was limited in both groups after correction for multiple comparisons. Together, these findings suggest that ES-based interventions incorporating concurrent movement observation may facilitate task-related sensorimotor rhythm modulation during the early stage of recovery following peripheral nerve reconstruction. The comparable alpha suppression observed under both ES conditions is consistent with a possible contribution of ES-induced proprioceptive input, although the independent effects of ES and action observation cannot be distinguished in the present study.

Based on Jeannerod’s motor simulation theory (27), action observation and MI could covertly activate the motor cortex as the effects of actual movement execution with similar premotor-parietal cortical networks (28). MI is primarily a conscious control through the top-down neural network, while action observation is a subconscious control involving a sensorial, perceptual, and affective bottom-up network. These interventions have been increasingly used to improve functional recovery after stroke (29, 30) and alleviate the effects of immobilization in individuals with transient sensorimotor deprivation (e.g., orthopedic trauma) (31). However, the present study did not observe significant alpha rhythm modulation during MI in individuals following peripheral nerve reconstruction after FDR correction. Lu et al. also found difficulty activating the supplementary motor cortex in patients with brachial plexus injury within 1 year, as shown by functional magnetic resonance imaging (32). This finding may suggest reduced engagement of sensorimotor networks during kinesthetic MI following peripheral nerve reconstruction.

Previous studies have suggested that reduced use of the affected limb and altered afferent input may contribute to contralateral sensorimotor cortical reorganization following peripheral nerve injury (6). Increased high-beta activity has been associated with endogenous top-down attentional modulation and task prediction processes (33). Although reduced afferent input has been proposed as one contributor to cortical reorganization and altered sensorimotor representations after peripheral nerve injury, the present study did not directly test this mechanism. Exploratory analyses using Semmes–Weinstein sensory scores showed no statistically significant associations with alpha modulation measures. A moderate negative association was observed between sensory status and MI-related high-beta modulation (*ρ* = −0.606, *p* = 0.084), although this finding should be interpreted cautiously given the small sample size. Therefore, the contribution of altered sensory input to MI-related cortical modulation remains speculative and warrants further investigation using larger cohorts and objective neurophysiological measures.

Combining neuromuscular ES with mirror therapy is an innovative approach that integrates both peripheral and central stimulation. This strategy has shown functional improvements in patients with strokes (34) or peroneal nerve injury (35). Our findings confirmed significant alpha RP suppression during ES combined with MVF, compared to the resting state, supporting its potential to modulate sensorimotor cortical activity.

However, the absence of a significant difference in alpha rhythm suppression between ES with and without MVF suggests that the addition of mirror visual feedback did not further enhance task-related sensorimotor cortical modulation beyond that observed during ES with concurrent action observation. Because both ES conditions required participants to observe movements performed by the unaffected hand, the present experimental design does not permit the independent contribution of electrical stimulation to be isolated. Nevertheless, the comparable alpha suppression observed in both ES conditions is consistent with the possibility that proprioceptive afferent input generated by ES contributes to sensorimotor cortical modulation when combined with action observation. This observation is particularly intriguing given the “invisible block” condition used in the present study and the impaired sensory feedback in patients with nerve reconstruction. Mechanical tension induced by electrically evoked muscle contractions may activate muscle mechanoreceptors and provide ascending proprioceptive signals that facilitate sensorimotor cortical activation, although this mechanism cannot be confirmed by the present study. These findings highlight a potential role of ES-induced proprioceptive input, acting together with action observation, in modulating sensorimotor cortical activity, and warrant further investigation.

Although low-beta modulation did not remain statistically significant after FDR correction, a trend toward increased low-beta power in the contralateral sensorimotor cortex was observed during ES with sham MVF. This finding may indicate that ES-generated proprioceptive input contributes to sensorimotor processing when combined with concurrent observation of movement, even in the absence of visual feedback. ES has been reported to modulate blood cytokines and increase ATP consumption, mimicking active exercise to promote axon regeneration and prevent muscle atrophy (15). Our finding aligns with a previous study (36) proposing that muscle contractions contribute to ascending sensory pathways via low beta oscillations. Although another study (37) reported reduced beta band power following ES relative to sham stimulation, differences in stimulation parameters and the presence of visible muscle contractions in the current study may account for the discrepancy. Because action observation was incorporated into both ES conditions, the present findings cannot distinguish the relative contributions of proprioceptive input and visual observation. Nevertheless, they support the hypothesis that ES-induced proprioceptive afferent signals may represent an important source of ascending sensorimotor information following peripheral nerve reconstruction.

As for ES combined with MVF, participants were instructed to execute movement from a mirror reflection of movement in the dominant or unaffected hand which induced action observation in a first-person prospection. Some studies revealed the impact of ES incorporated with action observation and MI on corticospinal activation up to 30 min after the intervention (14, 38). Additionally, prominent mu rhythm suppression was shown when ES was coupled with MI (39). In the present study, significant alpha rhythm suppression was observed during both ES combined with MVF and ES with sham MVF. Although the present design does not allow the independent contributions of action observation and ES-induced proprioceptive input to be distinguished, the mirror-mediated first-person visual feedback provided by MVF may further facilitate multisensory integration within the sensorimotor network. Given the absence of significant alpha modulation during MI in patients with peripheral nerve reconstruction, the combined sensorimotor inputs provided by ES and MVF may help compensate for reduced cortical engagement during MI. Future studies incorporating an ES-only condition will be required to clarify the relative contributions of proprioceptive input, action observation, and MVF.

Mirror therapy has been widely applied in neurorehabilitation to improve motor function following stroke (29) and to alleviate limb pain (8). Its underlying mechanism is thought to involve the establishment of functional connectivity between the bilateral motor cortices (40), which is crucial for maintaining the integrity of the sensorimotor cortex after peripheral nerve injury, such as traction or compression injuries. Although MVF-related cortical modulation has also been reported in stroke populations, the underlying neurophysiological context differs from that of peripheral nerve injury. In stroke, MVF is thought to facilitate recovery by rebalancing interhemispheric activity and modulating cortical excitability within damaged central motor networks (41, 42). Accordingly, several stroke EEG studies have demonstrated bilateral sensorimotor activation and modulation of interhemispheric balance during MVF. In contrast, the present study was designed *a priori* to evaluate task-related modulation within the contralateral sensorimotor cortex, which directly corresponds to the reconstructed limb and has been the primary region of interest in studies of peripheral nerve injury. Therefore, bilateral hemispheric comparisons were beyond the predefined scope of the present analyses. In the present study, ES combined with MVF was associated with enhanced modulation of sensorimotor alpha rhythms compared with MI alone following peripheral nerve reconstruction. While MI alone showed limited alpha modulation in patients, ES-based interventions consistently elicited sensorimotor alpha suppression. These findings suggest that ES combined with MVF may engage sensorimotor networks more effectively than MI alone during early neural recovery. However, because the present study did not separate the independent contributions of ES, action observation, and MVF, the underlying mechanisms should be interpreted cautiously. Therefore, MVF in patients with peripheral nerve reconstruction may contribute to sensorimotor integration through neurophysiological mechanisms that differ from those proposed in stroke rehabilitation. The interhemispheric mechanisms underlying these effects remain to be further elucidated.

Consistent with the present findings, Chen et al. provided evidence of neuroplastic effects following mirror therapy in patients with peripheral nerve injury (7). The previous study demonstrated increased activation in the multimodal association cortices during motor tasks, along with improved motor function following a 12-week mirror therapy intervention in patients with median or ulnar nerve injuries. Similarly, Rizzo et al. observed MVF-induced alpha event-related desynchronization in bilateral sensorimotor cortices during unilateral finger movements (12). In the present study, significant alpha rhythm suppression was found in both healthy individuals and nerve-injured patients during ES, regardless of whether MVF or sham MVF was used. These findings suggest that visual observation through mirror reflection facilitated by ES can activate the contralateral sensorimotor cortex both in healthy individuals and nerve-injured patients.

From a clinical perspective, these findings support the use of multimodal rehabilitation strategies that combine peripheral mechanoreceptor stimulation and visual feedback. Early after peripheral nerve reconstruction, voluntary motor activation may be limited due to incomplete reinnervation. In such cases, combining ES with MVF may provide additional sensory input that facilitates engagement of sensorimotor cortical networks. This approach may help promote cortical reorganization and support functional recovery during the early stages of neural regeneration.

Several limitations should be considered when interpreting these findings. First, the sample size was relatively small in the patient group, reflecting the limited availability of individuals within the early postoperative period after peripheral nerve reconstruction. Given the small patient sample, the non-significant MI finding should be interpreted cautiously. Although the effect did not reach statistical significance after FDR correction, the regression coefficient (B = −5.81) and confidence interval indicated a direction of change similar to that observed in healthy participants. Therefore, the possibility of a Type II error cannot be excluded. Second, participants following peripheral nerve reconstruction presented heterogeneous injury characteristics, including variations in nerve type, injury level, and surgical procedures. Objective measures of injury severity and reinnervation, such as nerve conduction studies or electromyography, were not systematically collected. Although exploratory analyses did not reveal significant associations between Semmes–Weinstein scores and EEG modulation measures, Semmes–Weinstein testing may not fully reflect the extent of peripheral nerve regeneration or cortical reorganization. These findings should therefore be interpreted cautiously, particularly given the small sample size. Third, although all participants demonstrated adequate imagery ability on the KVIQ-10 and were visually monitored during testing, the quality of MI during individual trials could not be objectively verified. Fourth, the present study focused on short-term task-related modulation of sensorimotor rhythms; therefore, longitudinal studies are required to determine whether these neural changes translate into long-term functional improvements. In addition, because both ES conditions incorporated observation of movements performed by the unaffected hand, the present design could not isolate the independent contributions of ES, action observation, and MVF. Therefore, the observed cortical modulation should not be attributed solely to ES. Moreover, resting-state EEG was recorded only once at the beginning of the experimental session and served as the baseline for all subsequent task conditions. Although the order of the three task conditions was randomized across participants to minimize potential order effects, changes in arousal, vigilance, or time-on-task may still have influenced alpha-band modulation during later conditions. Future studies incorporating a stimulation-only condition without visual observation, repeated baseline recordings, and formal evaluation of order effects are warranted to clarify these methodological issues.

Future studies incorporating standardized neurophysiological assessments, bilateral EEG analyses or hemispheric lateralization indices, and larger samples with longitudinal designs are needed to clarify the peripheral–central interaction underlying cortical reorganization, and the role of multimodal interventions in promoting cortical reorganization following peripheral nerve reconstruction. Longitudinal controlled studies are also required to determine whether ES combined with MVF provides superior functional outcomes compared with ES alone.

## 5 Conclusion

In conclusion, alpha modulation during MI was less evident in patients following peripheral nerve reconstruction. Given the limited sample size, the non-significant MI finding should be interpreted cautiously. In contrast, ES-based interventions were consistently associated with sensorimotor alpha suppression. In particular, ES combined with MVF demonstrated greater modulation of sensorimotor alpha rhythms than MI alone. These findings suggest that multimodal sensorimotor stimulation may facilitate task-related cortical engagement during the early stage of recovery following peripheral nerve reconstruction. However, the present findings reflect acute task-related neural modulation and do not establish superior long-term functional outcomes.

## Data Availability

The original contributions presented in the study are included in the article/Supplementary material, further inquiries can be directed to the corresponding author.
