# Supplementary material for: Differential modulation of sensorimotor alpha rhythms by motor imagery, electrical stimulation, and mirror visual feedback following peripheral nerve reconstruction: an EEG study
Source: Front Neurol. 2026 Aug 21;17:1836039. doi: 10.3389/fneur.2026.1836039 (PMC13541493; doi:10.3389/fneur.2026.1836039)
Supplement: Supplementary file 1 [file Supplementary_file_1.docx]

Supplementary Material

**Supplementary Table 1.** Baseline-normalized changes in sensorimotor relative power (ΔRP) across experimental conditions.

| Frequency  Band | Condition | Healthy (n=21) | | |  | Patients (n=9) | | |
| --- | --- | --- | --- | --- | --- | --- | --- | --- |
|  |  | Mean ± SE | B (95% CI) | FDR-adjusted *p* value |  | Mean ± SE | B (95% CI) | FDR-adjusted *p* value |
| Alpha | MI | -0.18 ± 0.04 | 0.07 (0.02, 0.11) | **0.009** |  | -0.13 ± 0.11 | 0.12 (0.00, 0.23) | 0.090 |
|  | ES | -0.20 ± 0.05 | 0.04 (-0.03, 0.11) | 0.278 |  | -0.19 ± 0.04 | 0.06 (-0.04, 0.15) | 0.262 |
|  | MVF | -0.24 ± 0.05 | Reference | － |  | -0.25 ± 0.07 | Reference | － |
| Low beta | MI | 0.31 ± 0.27 | 0.01 (-0.15, 0.17) | 0.944 |  | 0.11 ± 0.06 | 0.02 (-0.16, 0.20) | 0.854 |
|  | ES | 0.23 ± 0.18 | -0.08 (-0.19, 0.04) | 0.431 |  | 0.13 ± 0.05 | 0.03 (-0.10, 0.15) | 0.854 |
|  | MVF | 0.31 ± 0.22 | Reference | － |  | 0.10 ± 0.11 | Reference | － |
| High beta | MI | 0.36 ± 0.22 | 0.07 (-0.11, 0.24) | 0.669 |  | 0.33 ± 0.19 | 0.28 (0.00, 0.56) | 0.099 |
|  | ES | 0.26 ± 0.15 | -0.03 (-0.18, 0.11) | 0.669 |  | 0.13 ± 0.09 | 0.08 (-0.12, 0.28) | 0.453 |
|  | MVF | 0.29 ± 0.17 | Reference | － |  | 0.05 ± 0.07 | Reference | － |

Data are presented as mean ± standard error (SE). Generalized estimating equation (GEE) models were used to estimate condition effects relative to the mirror visual feedback (MVF) condition (reference). Regression coefficients (B), 95% confidence intervals (CI), and Benjamini–Hochberg false discovery rate (FDR)-adjusted *p* values are reported for each condition. FDR correction was performed separately within each frequency band and group. Significant adjusted *p* values (p < 0.05) are shown in bold. MI, motor imagery; ES, electrical stimulation with sham mirror visual feedback; MVF, electrical stimulation combined with mirror visual feedback.

**Supplementary Figure 1.** EEG spectral power of one healthy participant, with right right-hand dominant, and one patient with right median nerve injury. (A) The alpha band of the healthy subject. (B) The alpha band of the patient. (C) Low beta band of the healthy subject. (D) The low beta band of the patient. (E) High beta band of the healthy subject. (F) High beta band of the patient. MI, motor imagery; ES, electrical stimulation combined with sham mirror visual feedback; MVF: electrical stimulation combined with mirror visual feedback.
